# Targeting folliculin to selectively inhibit mTORC1: a promising strategy for treating nonalcoholic fatty liver disease

**DOI:** 10.1038/s41392-022-01111-x

**Published:** 2022-08-09

**Authors:** Yan Ling, Yanpeng Li, Liang Li

**Affiliations:** 1 Changzheng Hospital, Naval Medical University, Shanghai, 200003 People’s Republic of China; 2 National Center for Liver Cancer, Naval Medical University, Shanghai, 201805 People’s Republic of China

**Keywords:** Metabolic disorders, Gastrointestinal diseases

In a recent study published in *Science*, Bridget S. Gosis et al. demonstrate that selective inhibition of the mammalian target of rapamycin complex 1 (mTORC1) signaling through deletion of the RagC/D guanosine triphosphatase-activating protein folliculin (FLCN) in mice enhances activation of transcription factor E3 (TFE3) in the liver and protects against nonalcoholic fatty liver disease (NAFLD).^1^

NAFLD is the primary cause of chronic liver disease and affects one-fourth of the adult population worldwide.^2^ In the clinic, there are currently no FDA-approved medicines for the targeted therapy of NAFLD. Intriguingly, targeting FLCN to inhibit mTORC1 for the treatment of NAFLD does not affect other substrates of mTORC1 and is, therefore, able to avoid unanticipated feedback loops and undesired side effects.^1^

**Fig. 1:**
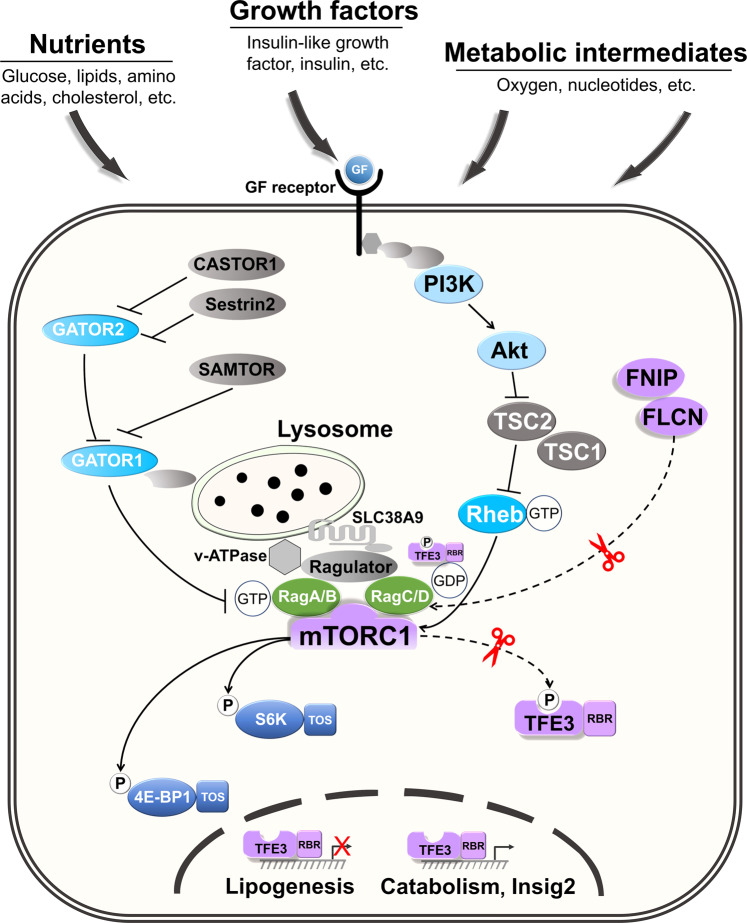
Canonical and noncanonical mTORC1 signaling based on substrate recruitment and phosphorylation distinctions. mTORC1 is a signaling hub that integrates numerous upstream inputs, including nutrients, growth factors, and metabolic intermediates. Rags is needed for the physical recruitment of mTORC1 to the lysosomal membrane, which is required for mTORC1 activation. The action of mTORC1 on its canonical substrates S6K and 4E-BP1 is regulated by the binding of growth factors and their receptors, which promote the activity of PI3K-Akt-TSC-Rheb signaling axis to activate mTORC1. Instead, the phosphorylation of TFE3 is controlled by FLCN-RagC/D-mTORC1 signaling axis. S6K and 4E-BP1 both have a TOS motif, whereas TFE3 has a Rag-binding region in its structure. This is the mechanism underlying the distinct phosphorylation behaviors of these two mTORC1 substrate classes. GF growth factor, P phosphorylation, GTP guanosine triphosphate, GDP guanosine diphosphate, TOS TOR signaling motif, RBR Rag-binding region

Multiple studies have demonstrated that mTORC1 is a focal signaling node that incorporates numerous upstream stimulations, such as nutrients (glucose, lipids, amino acids, cholesterol, etc.), growth factors (insulin-like growth factor, insulin, etc.), and metabolic intermediates (oxygen, nucleotides, etc.),^3,4^ thus collaborating the reaction of cellular metabolism to external cues (Fig. 1).

mTORC1 function is highly reliant on its physical recruitment to the lysosomal membrane, which is dependent on the activity of Rag GTPase heterodimeric complexes (RagA/B and RagC/D) and the pentameric lysosomal complex Ragulator.^4^ RagA/B must be in the GTP-bound state for mTORC1 to be recruited to the lysosomal surface. RagC/D contributes to dimer formation, although its GTP/GDP-binding state is less important for mTORC1 lysosomal recruitment. The sophisticated machinery of trimeric GATOR1 (GTPase-activating protein activity toward Rags 1), which functions as a GAP (GTPase-activating protein), precisely modulates the nutrient-dependent transition of RagA/B activity. The GATOR2 complex counteracts the activities of GATOR1. Several cytoplasmic amino acid sensors, including Sestrin2, CASTOR1, and SAMTOR, sense intracellular amino-acid levels and transmit their information to mTORC1 via GATOR complexes. The solute carrier SLC38A9 facilitates intralysosomal amino acid level sensing, and the lysosomal v-ATPase complex is necessary for the preservation of the lysosomal proton gradient^4^ (Fig. 1).

Lysosomal recruitment of mTORC1 is essential for mTORC1 activation by the small GTPase Rheb (Ras homolog, mTORC1 binding). Growth factors (GFs) binding to their receptors can activate PI3K-Akt signal and subsequently block the GAP activity of the Rheb inhibitor TSC (trimeric tuberous sclerosis complex), resulting in Rheb conversion to the functional GTP-bound state. Rheb binding to mTORC1 induces profound conformational changes that result in the activation of the canonical mTORC1 substrates S6 kinase (S6K) and eukaryotic initiation factor 4E-binding protein 1 (4E-BP1).^3,4^ Briefly, in response to upstream stimulations, activation of mTORC1 and canonical substrate phosphorylation need nutrient-stimulated Rag activity and GF-dependent Rheb activation (Fig. 1).

Recent evidence supports the existence of noncanonical signaling for selective mTORC1 regulation, which is based on a recently found mechanism of mTORC1 substrate recruitment and phosphorylation. A growing body of research indicates that nutrient replenishment triggers FLCN:FNIP (folliculin: folliculin-interacting protein) separation from the lysosomal surface and boosts its GAP activity toward RagC/D, which transforms GTP-RagC/D into GDP-RagC/D and facilitates the activation of mTORC1. Excitingly, FLCN clearance in a variety of cells suppresses mTORC1-mediated phosphorylation and inactivation of the transcription factor E3/B (TFE3/B) family without altering mTORC1-driven phosphorylation of its canonical substrates S6K and 4E-BP1.^1,4^ This selective phosphorylation of mTORC1 has been clarified by recent studies in which researchers discovered that S6K and 4E-BP1 comprise a TOR signaling (TOS) motif, a five amino acid region directly recognized and linked by mTORC1 through its subunit Raptor, thereby enabling substrate recruitment and phosphorylation. Instead of the TOS motif, TFE3/B possess a Rag-binding region (RBR) in their N-terminal regions, allowing them to engage with Rags in this way. TFE3/B interaction is only possible when RagA/B and RagC/D are connected to GTP and GDP, respectively. Upon that basis, TFE3/B phosphorylation is acutely susceptible to upstream inputs that affect Rag activity, such as nutrients, but is insensitive to other mTORC1-activating stimulations, such as growth factors. Among these regulatory mechanisms, RagA/B activity is essential for mTORC1 lysosomal localization and, as a result, for its action on all substrates. Furthermore, RagC/D activity is strictly required for TFE3/B recruitment and phosphorylation, and it serves a secondary role in mTORC1 lysosomal recruitment and phosphorylation of the canonical substrates S6K and 4E-BP1.^4^ Because of this, it is possible to separate canonical and noncanonical signaling downstream of mTORC1 by modulating FLCN (Fig. 1).

mTORC1 has the ability to trigger selective responses to a wide variety of stimuli, which has been demonstrated to be related to some physiological and pathological conditions. Suppression of FLCN in the liver by Bridget S. Gosis et al. has been shown to protect mice against NAFLD and partially reverse these processes, indicating that FLCN could be a therapeutic target for NAFLD.^1^ Hepatocyte-specific FLCN deletion in mice preferentially inhibits mTORC1-mediated phosphorylation of TFE3 and promotes its entrance into the nucleus, but has a modest effect on the canonical substrates S6K and 4E-BP1. Targeting this FLCN:mTORC1:TFE3 arm appears to simultaneously induce lipid consumption programs and suppress de novo lipogenesis. When TFE3 is released into the nucleus, Insig2 is activated to prevent the proteolytic processing of SREBP-1c, enhance the interaction of TFE3 with chromatin close to SREBP-1c, and further inhibit adipogenesis-related genes (Fig.1).

As a multi-pathogenic disease, NAFLD affects a wide range of intrahepatic cell types and involves an intricate web of signal transduction pathways.^5^ Approximately 500 different functions are attributed to the liver when working with other systems and organs. Therefore, how to properly target NAFLD without disrupting other liver processes remains a key difficulty. mTORC1 governs hepatic lipid production and catabolism, but it also regulates numerous other hepatocyte pathways; hence, inhibiting mTORC1 could result in unwanted feedback loops. This study^1^ permitted selective control of liver cell metabolism by the FLCN:mTORC1:TFE3 arm and exhibited very few undesired effects in the treatment of NAFLD, thus opening up a new therapy option for NAFLD.
